# Apendicitis Complicating Pregnancy

**Published:** 1918-03

**Authors:** Aimé Paul Heineck

**Affiliations:** Professor of Surgery, Chicago College of Medicine and Surgery, Chicago


### BUFFALO MEDICAL JOURNAL

Yearly Volume 73 MARCH, 1918 Number 8

### ORIGINAL ARTICLES

**The right is reserved to decline papers not dealing with practical medical and surgical subjects, and such as might offend or fail to interest readers. Contributors are solely responsible for opinions, methods of expression and revision of proof.**

### Appendicitis Complicating Pregnancy

By AIMÉ PAUL HEINECK, M. D.,

Professor of Surgery, Chicago College of Medicine and Surgery, Chicago.

Appendicitis attacks all ages and both sexes. Though distinctly a surgical disease, it is also of great practical interest to gynecologists, obstetricians and general practitioners.

The frequency of appendicitis in the female pregnant or non-pregnant is under-estimated and it's significance not fully appreciated. It is often over-looked, misdiagnosed and therefore improperly treated. The autopsy findings often bring the first intimation of the true cause of the clinical picture.

To serve our fellow practitioners, we collected, analyzed and studied the original reports of all the operated cases of appendicitis occurring during pregnancy, that are to be found in the French, English and German medical literature from 1900 to 1915, inclusive, and also some unpublished personal cases. Cases reported with insufficient data were not considered.

The subject will be discussed under the following subheads:

1. Incidence.
2. Etiology.
3. Combined appendicitis and extra-uterine pregnancy.
4. Pathology.
5. Coexisting conditions.
   Influence of pregnancy upon appendicitis.
   Influence of appendicitis upon pregnancy.

6. Diagnosis.
7. Differential diagnosis.
    (a) Maternal.
    (b) Fetal.
8. Prognosis.
9. Treatment.
    (a) Prophylaxis.
    (b) Indication for operation.
    (c) Operative.
10. Post operative sequelae.
11. Summary.

### INCIDENCE.

During the child bearing age, woman is at no time exempt from attacks of appendicitis. In forty-six of our selected cases, the age is not stated. The remaining patients were at time of operation:

| | |
|---|---|
| Under 18 years | 3 cases |
| 18-20 years inclusive | 13 cases |
| 21-25 years inclusive | 33 cases |
| 26-30 years inclusive | 42 cases |
| 31-35 years inclusive | 23 cases |
| 36-40 years inclusive | 12 cases |

One patient forty-two years.

The condition occurs in primiparae and multiparae; in first early and late pregnancies; in single and twin pregnancies. Appendicitis can coexist with other disease processes to which it may be primary, secondary or coincidental.

In the cases forming the basis of this article, there are noted thirty primiparae, twenty deutiparae, thirty-seven multiparae.

The number of previous pregnancies if there were any, is not stated in eighty-three cases. Appendicitis occurs at all periods of gestation. In some cases, the disease antedated pregnancy; some cases were operated early with reference to onset of symptoms; some late. It is recorded that operation was indicated and performed:

| | |
|---|---|
| During the first three months of gestation | 40 times |
| From 4-6 months, inclusive | 60 times |
| From 7-9 months, inclusive | 28 times |
| Period of gestation not stated | 45 times |

### ETIOLOGY.

The etiology of appendicitis in a pregnant woman is the etiology of appendicitis in the non-pregnant woman. It is the belief of many clinicians that gestation does not exert any influence, good or bad, upon the normal appendix.

Appendicitis is primary or secondary; it may be secondary to disease of the uterine adnexa just as inflammatory diseases of the tube and ovary may be secondary to an appendicitis. Recurrent attacks of appendicitis may be precipitated by pregnancy, labor or puerperium. Pregnancy can provoke acute inflammatory disturbances in an appendix bound down by dense adhesions or containing a foreign body, one or more fecal concretions, or worms. The appendicitis complicating pregnancy may be the patient's first attack. It may have been preceded by one, two, three, or more attacks of greater or less severity.

COMBINED APPENDICITIS AND EXTRA UTERINE-PREGNANCY.

In some of the reported cases in which appendicitis and ectopic pregnancy were associated, it was not determined which of the two conditions antedated the other; which was primary and which was secondary.

When an appendicitis precedes a tubal pregnancy in which it apparently plays an etiological role, the anatomical changes frequently evolute as follows:

1. Appendicitis.
2. Peri-appendicitis.
3. Peri-adnexitis.
4. Formation of inflammatory adhesions interfering with tube mobility and tube function and producing tubal malformation.
5. Tubal pregnancy.

All these conditions favor the ectopic implantation of fertilized ova. Appendicitis may hasten tubal abortion through local infection, through general intoxication, may lead to suppuration of homatoceles of fetal cysts.

To differentiate appendicitis from extra-uterine pregnancy is at times difficult. In the unruptured state, the pregnant tube gives symptoms analogous to those of chronic appendicitis. An infected hematocele presents the signs of suppurative pelvic peritonitis. Peritoneal hemorrhage due to a ruptured tubal gestation sac has symptoms closely resembling a diffuse septic peritonitis. Positive Abderhalden test, absence of fever, vaginal hemorrhage, symptoms of internal hemorrhage will point to tubal pregnancy. It is interesting to make an exact diagnosis but as both diseases are surgical affections exposing mother and fetus to serious dangers, the watchword in both conditions should be early operation. Appendicitis calls for prompt operative treatment; extra-uterine pregnancy is an emergency condition calling for immediate ablation of the ectopic fetal sac.

In all the cases of appendicitis and extra-uterine pregnancy herein considered, twelve in number, operation gave excellent results. The findings differed in nature and consequently the operative procedures varied in extent in the different cases.

PATHOLOGY.

Acute and chronic inflammations of the appendix involve the organ, in part or in its entirety, and are associated with catarrhal, fibrinous, sero-fibrinous, sero-purulent, or purulent exudates present in the cavity of the appendix, in it's wall, or around it. The inflammatory process may be limited to the mucous membrane, may involve part of, or the entire thickness of the appendical wall.

The appendix vermiformis may be partly or wholly intra- or extra-peritoneal. A retro-peritoneal or extra-peritoneal appendix the seat of suppurative inflammation gives rise to retro-peritoneal or extra-peritoneal pus collections. Adhesive inflammation may lead to permanent fixation of the appendix, to one or more abdominal viscera normal or pathologic, to the abdominal parietes, or to both. Inflammatory adhesions involving the tube, may angulate it, constrict it; may interfere with tubal mobility and tubal function; may change it's course and play a fairly important role in the etiology of sterility. The appendix, during a 280 day pregnancy, may touch every organ of the abdomen. Pus in quantities, large or small, may be present within the cavity of the appendix, in it's wall or around it. Acute suppurative inflammation of the uterus and tubes may be set up by direct extension from an acutely inflamed appendix. The walls of appendiceal or peri-appendiceal abscesses are formed in part by one or more of the following organs: uterus, adnexa, omentum, intestine, small or large, etc. An appendicular abscess may bulge into the posterior cul-de-sac, may open spontaneously into the uterus, vagina, rectum.

The inflammation proceeded to the state of gangrene in twenty-four cases; in eleven of these cases one or more perforations were present. The gangrene may be limited to the mucous membrane, may affect the entire appendiceal wall or the entire organ. Any part of the organ, tip, middle, base, may be gangrenous. Fecal concretions, one or more, were present in thirteen appendices. It is easy to understand how inflammation migrates from the appendix to the Fallopian tube, to the pregnant uterus, etc. Pelvic inflammatory processes extending by continuity or contiguity of tissue, occur in the pregnant as well as the non-pregnant. Distal pus collections are due to metastases by way of the lymph or blood channels. In the ulcerative type of inflammation the ulcer extends in depth and in surface area; when all the coats of the appendix have been burrowed through, a perforation results. The apex, the base, or any other part of the appendix may be the seat of the perforation.

### COEXISTING PATHOLOGICAL CONDITIONS.

Coexisting pathological conditions are primary or secondary to the appendiceal inflammation or merely coincidental, bearing no relation of cause or effect to it. It is not uncommon for appendicitis in the female to be complicated by or associated with tubal and ovarian diseases: salpingitis, pyosalpinx, hydrosalpinx, ovarian abscess, tube-ovarian abscess, para-metritis, etc. Close anatomical association of the appendix with the uterus and the adnexa explains the frequent simultaneous involvement of these organs in disease processes.

### INFLUENCE OF PREGNANCY UPON APPENDICITIS.

Upon a normal appendix, gestation has little or no influence. Upon an appendix, the seat of previous or latent disease, pregnancy exerts an unfavorable influence. It can intensify an existing inflammation. It may cause a previous inflammation to recur. In view of this possibility, many of our best clinicians recommend and practice the removal of the appendix in women married or about to be married who have had one or more attacks of appendicitis non-operatively treated.

The pregnant uterus as it ascends in the abdomen commonly displaces the coecum and the appendix from below up, from right to left and from behind forward. In enlarging, the uterus may stretch existing inflammatory adhesions; it may displace, twist, and kink the appendix and thereby whip into activity latent appendicular infections. Pregnancy is a serious complication of appendicitis, 1. When the appendix is adherent to the uterus. 2. When it is the seat of an inflammation, perforative, gangrenous or suppurative in type. 3. When it's inflammation leads to abscess-formation, near or distal. 4. When the uterus forms part of the wall of an appendicular, peri- or para-appendicular abscess. In the afore mentioned conditions, adhesions may be torn, abscesses may be ruptured by the enlarging uterus.

### INFLUENCE OF APPENDICITIS ON PREGNANCY.

Appendicitis is a menace to the mother's life, it is a menace to the gestation. The danger increases with the advance of gestation and is most marked after the fourth month. Infection can and does spread from the appendix to the genital organs by way, 1. Of the peritoneum (localized or diffuse peritonitis). 2. Of the appendico-ovarian ligament. 3. Of adhesions existing between the uterus and a perityphilitic pus focus. 4. Of the Fallopian tube.

Even a mild case of appendicitis may lead to a plastic peritonitis closing permanently the lumina of both tubes. From inflammatory adhesions may result dysmenorrhea, subinvolution, sterility through inflammatory closure of tubal ostia, habitual abortion, extra-uterine pregnancy, a tendency to uncontrollable vomiting, etc.

Appendicitis in the pregnant state may or may not terminate pregnancy. The prognosis is good as to non-interruption of pregnancy. 1. When the appendix does not hang in the small pelvis. 2. When the inflammation is limited to the appendiceal mucosa. 3. When it does not extend beyond the appendiceal wall. 4. When the appendiceal abscess or periappendiceal abscess is small.

Premature termination of gestation either by fetal death, fetal expulsion or both may be caused by, 1. Sequels of previous appendicitis, acute or chronic; inflammatory adhesions, old or recent, preventing uterine expansion. 2. Infection from the appendix extending through the tubes to the uterus and it's contents. 3. Infection reaching the placenta through lymphatic and vascular channels. 4. Metastatic inflammation of the placenta disturbing it's circulation. 5. Local irritation. 6. Fatal effect of hyperpyexia upon ovum.

The further pregnancy is advanced the greater the danger of abortion after operation. The chance of abortion after early operation is very small indeed for the operation is then done before an extensive inflammation has involved the uterus or an abscess rendered the patient septic. Tendency to abortion is small in clean cases as in this type the operative manipulation is reduced to a minimum.

In 173 cases of appendicitis herein studied it is stated that abortion was artificially induced nine times and occurred spontaneously forty-nine times. Caesarian section was performed four times. Abdominal one, vaginal three.

In eighty-three cases, pregnancy was not interrupted by the operation. In seventeen cases, no definite statement is made.

DIAGNOSIS.

Appendicitis is not as frequently misdiagnosed as formerly. Increased familiarity with the condition enables us to make an earlier and a more timely diagnosis. It is an established fact that the morbidity and mortality of this disease can be lessened if it be diagnosed and operated, before the advent of complications, perforation, gangrene abscess formation, peritoneal involvement, etc. The diagnostic difficulties increase with the advance of gestation and persist during the puerperium.

The symptomatology of appendicitis in the pregnant is the symptomatology of the disease in the non-pregnant. Nevertheless, the recognition of the condition is made more difficult by various factors. One or more of the cardinal symptoms may be lacking. The symptoms and signs may not be sufficiently pronounced to lead to careful investigation or may be classed among the various disturbances incident to pregnancy.

During pregnancy the abdominal walls are on the stretch; they lack the softness and pliability so essential to careful and satisfactory abdominal palpation. In very fleshy patients palpation does not give definite findings.

The seat of pain though always corresponding to the site of the inflamed appendix, may be abnormally high. The leukocyte count gives uncertain findings; at best, it has only relative or corroborative value.

Mistakes are less likely to occur by keeping in mind (a) that every pregnant woman is to be examined for physical defects; (b) That the history is all important; ask about previous attacks. (c) In gravid women, all attacks of indigestion associated with vomiting and fever should arouse suspicion and command a careful examination of the abdomen. (d) Right iliac pain unassociated with uterine contractions should lead one to think of appendicitis. (e) Deep seated retro-coecal and other abscesses may be detected by rectal examination. (f) Peri- or para-typhilitic abscesses may be detected by vaginal examination.

In a pregnant woman, acute abdominal pain of a sudden onset, at first diffuse and then remaining localized to the right iliac fossa, suggests appendicitis; more so if the patient gives the history of previous attacks.

### DIFFERENTIAL DIAGNOSIS.

During gestation, many conditions simulate appendicitis. As most of these conditions demand operative relief, the resulting diagnostic mistakes are embarrassing and humiliating to the surgeon, but not commonly disastrous to the patient. In adnexal disease the pain and the objective findings are most always bilateral, while in appendicitis they are unilateral and the pain as a rule is more acute. Non-ruptured right tubal pregnancy simulates and is frequently diagnosed as chronic appendicitis. Rigidity and tenderness over McBurney's point are seldom marked in extra-uterine pregnancy. Intelligent interpretation of the clinical history and of the objective findings, furnished by a careful and thorough abdominal, rectal and vaginal examination helps one to arrive at a correct diagnosis. Abscesses in the pouch of Douglas due to perforative appendicitis have been wrongly attributed to primary uterine and tubal infection; right-sided parametritis due to the spreading of a retro-colic appendicitis has been diagnosed ordinary puerperal infection.

In pyelitis, uteritis, ureteric calculus of the right side one is guided by the urinary symptoms and findings. Hepatic colic has a sudden onset with pain in the right upper abdominal quadrant; this pain radiates toward the right shoulder and is usually apyretic. The pain of nephritic colic descends and radiates toward the external genitalia. In fecal impaction, the symptoms are less severe and yield to colonic injections and to laxatives.

In advanced pregnancy, the differential diagnosis between appendicitis and cholecystitis may prove difficult owing to the associated upward displacement of the coecum and appendix by the pregnant uterus.

### PROGNOSIS.

Pregnancy increases the severity and the fatility of appendicitis. Death may be due to intestinal obstruction, to perforation of the appendix, to heart failure, to peritonitis, or to sepsis. Recovery takes place through the gradual subsidence of symptoms; through the spontaneous rupture of an appendicular abscess externally, or into the gut, vagina, urinary bladder, uterus, or other hollow viscus.

The type and the acuity of the inflammation influence the prognosis. The prognosis is good if the changes in the appendix are slight, if the inflammation is limited to the appendiceal wall; if there be slight or no peritoneal involvement, if complications be absent. It is grave in gangrenous, perforative and suppurative appendicitis and in all cases complicated by abscess formation near or distal, or by diffuse peritonitis. The results for the mother and fetus are better, the less advanced the gestation, the less virulent and wide spread the inflammation, the earlier the operation. Maternal mortality of appendicitis in pregnancy increases from the fourth month on.

As far as the child is concerned, prognosis is absolutely good in cases of early operated appendicitis. Severe maternal appendicitis is exceptionally grave for the fetus, who succumbs either through infection or through interruption of pregnancy. In our cases, there were fifty-eight abortions; of these nine were induced and forty-nine were spontaneous. The spontaneous abortions gave seventeen maternal deaths and thirty-two recoveries. The induced abortion gave four maternal deaths and five recoveries.

### PROPHYLAXIS.

The cause of appendicitis is not known. Therefore, in the present state of our knowledge a discussion of the prophylaxis of appendicitis, of necessity must be and is incomplete, inadequate and inconclusive. The importance of constipation as an etiological factor in appendicitis is as yet undetermined.

We do know how to prevent appendicitis, but do we know how to lessen its morbidity and mortality. Some surgeons remove the appendix during the course of all laparotomies. The removal of a healthy organ because one is not certain that it will always remain free of disease is unnecessary, meddlesome, and contrary to the teachings of conservative surgery.

In all laparotomies for conditions other than appendicitis, if the patient's condition permits, the appendix should be examined and removed, 1. If it be abnormal in length, size or location. 2. If it be in close relation to a pedicle or denuded surface, left by operation. 3. If its cavity be partly or wholly obliterated. 4. If it be the seat of anatomic alterations, club-shaped, thickened, kinked, twisted, strictured, etc. 5. If it contain foreign bodies, fecal concretions, worms, etc. 6. If it be adherent, in part or in its entirety, to some normal or diseased contiguous organ or to the abdominal parietes. 7. If it be the sole content or one of the contents of a hernial sac. 8. If it be the seat of cystic, neoplastic or inflammatory disease.

Operations that contribute to the safety of a pregnant woman should be performed without hesitation.

### INDICATIONS FOR OPERATION.

Clinical cures obtained by medicinal measures are rarely anatomical cures. Starvation treatment is debilitating to the mother, is unfavorable to fetal growth. Perforation, abscess, general peritonitis, subdiaphragmatic abscess, thrombosis and embolism are possible results of expectant treatment. Better to remove too many appendices than too few. Be not deterred by the possibility of a difficult operation for the results of early operation are satisfactory and the mortality low.

Operate early in the attack and early in the course of pregnancy. As a general proposition, operation does not interrupt pregnancy. The triumphs of ovariotomy and hysterectomy in pregnancy are well known; in appendicitis operation is even more urgent. Accumulated instances are on record in which pregnant uteri have been operated upon, cauterized, etc., in which ovarian and other pelvic tumors have been removed without pregnancy being terminated. The high mortality of appendicitis in pregnant women is due to fatal temporization. Placental, uterine and peritoneal infections are such serious complications that one should if possible operate before the inflammatory process has taken place, before the onset of peritoneal or other complications.

Operate early in gestation. At that period the uterus is not large enough to be in the way. The operation is less diffcult; the tendency to the interruption of pregnancy is minimal and the percentage of maternal recoveries is higher. The danger of recurrence in the latter months of gestation calls for operation during the attack; if that be not feasible an interval operation should be performed as long before the labor as possible.

Operation in fifty cases of non-perforative appendicitis gave only one maternal death and seven abortions. In fifty-five cases of diffuse peritonitis secondary to appendicitis, there were forty-four maternal deaths, only one child was saved, all the others were born prematurely or died soon after birth from weakness, or the illness of the mother resulted fatally before the termination of labor.

### TREATMENT.

Interruption of pregnancy is not indicated; it increases the danger. Rest should be enjoined; during the operation, the uterus should be handled and exposed as little as possible, after the operation, opiates should be administered. In a clean case, the operative manipulation is slight. Artificial evacuation of the the uterus before laparotomy is indicated only when the child is dead or when there are appreciable signs of labor. If the uterus be artificially emptied before the seventh month, the child will be definitely lost and the patient not improved. By evacuating an appendiceal abscess before emptying the uterus one avoids flooding the free peritoneal cavity with pus. Operations for appendicitis are performed under local or general anaesthesia. Some operators resort to lumbar anaesthesia. Operate as rapidly as is consistent with thoroughness and th( patient's welfare.

The operation of election is appendectomy, the technique of which is little influenced by the presence of pregnancy. The same surgical principles are applicable in the pregnant as in the non-pregnant.

When in doubt as to whether the case is one of appendicitis, salpingitis, tubal pregnancy or other pathological conditions, use a supra-pubic median incision. This incision affords easy access to most of the pelvic contents and though it is not the incision of election for exposure of the appendix, it is a very serviceable incision. In cases of combined appendicitis and salpingitis, combined appendicitis and tubal pregnancy, combined uterine myoma and appendicitis, etc., the median infraumbilical incision should be employed.

In 125 of our cases the appendix was removed. In forty-three cases, it is not stated whether it was removed or not. In five cases, it was sought but not found, and therefore not removed. Each of these cases presented an abscess, which was evacuated and drained. If the appendix be imbedded in a mass of firm inflammatory adhesions, it can be removed by shelling it out of its peritoneal coat.

An appendiceal abscess should be opened at its point of maximal bulging; preferably through a cutaneous surface. If the appendix be not easily found, be content with incising the abscess, evacuating its contents and resorting to tube or gauze drainage. A subsequent operation will rarely be required to remove the appendix. Appendiceal abscesses have been opened and drained through the vagina. Appendiceal abscesses have also been opened through the rectum. These are exceptional procedures: methods of necessity, not of election.

The post operative treatment is that which is employed in the non-gravid modified only by a longer sojourn in bed, thereby giving time for firm consolidation of the operative wound.

### POST OPERATIVE COMPLICATIONS AND SEQUELAE.

In cases of such widely different nature as those herein studied, operated in different surroundings and by different operators, one is not surprised to find noted the occurrence of post operative complications and post operative sequelae. The danger of hernia development after timely operations for appendicitis is practically nil. The protection of the operative scar by the aid of adhesive plaster has been recommended. See that labor be not unduly prolonged.

Among the sequelae reported in these cases were four ventral hernias, three cases of diffuse peritonitis, thrombosis of femoral veins, phlebitis, subphrenic abscess, intestinal fistulae, etc.

### SUMMARY.

1. Appendicitis occurs at all ages and in both sexes. It presents to all medical men important diagnostic, prognostic and therapeutic features.

2. Appendicitis acute, or chronic, initial, relapsing or recurrent, primary or secondary, complicates pregnancy with greater frequency than is believed. It is the most important complication of pregnancy.

3. It occurs in single and twin gestations; in first, early and late pregnancies; in primiparae, deutiparae, and multiparae.

4. It occurs at all periods of the child-bearing age and at all periods of gestation. It complicates both intra- and extrauterine pregnancies and can coexist with other disease processes to which it may be primary, secondary or coincidental.

5. Gestation exerts no untoward influence upon the normal appendix. It can and frequently does aggravate existing, or determine new inflammatory disturbances in appendices deviating from the normal in form, length, mobility, location, etc., in appendices bound down by adhesions or the seat of inflammatory or other degenerative changes. Pregnancy does not relieve the dangers of appendicitis, but aggravates them.

6. Appendicitis and uni or bilateral tubal pregnancy are frequently mistaken for each other. They may occur simultaneously or consecutively, may be either primary or secondary to, or independent of each other.

7. In appendicitis, in ectopic pregnancy and in combined appendicitis and ectopic pregnancy, of obscure symptomatology, it matters not whether you are certain or in doubt as to the real diagnosis, early and timely operative treatment is imperatively indicated.

8. During gestation, every type of appendicitis may occur: adhesive, catarrhal, gangrenous, ulcerative, obliterative, perforative and suppurative.

9. Appendicitis with adhesion formation is of great significance because adhesions of inflammatory origin can (a) incarcerate the pregnant uterus in the pelvis and mechanically hinder the enlargement of the uterus, (b) impair the contractibility of the uterus, (c) interfere with uterine labor contractions, (d) entail subinvolution, (e) induce sterility, (f) disturb tubal and ovarian integrity of function and of structure, (g) determine ileus, (h) produce abortion, and, (i) lead to extra-uterine pregnancy.

10. Chief among the coexisting pathological conditions! noted in appendicitis are simultaneous or consecutive inflammation of the uterus, tubes or other pelvic organs. The close anatomical relations existing between the appendix and the pelvic organs explain their frequent association in disease processes.

11. Appendicitis has a greater morbidity and a higher mortality in the pregnant than in the non-pregnant, operated or non-operated. It may terminate pregnancy.

12. The symptomatology of appendicitis in the pregnant is the same as in the non-pregnant. The clinical picture however, is blurred by the coexisting symptoms of pregnancy. Diagnostic mistakes may be lessened by keeping in mind that appendicitis occurs in pregnant women; that a history of previous attacks during the same or previous pregnancies can frequently be elicited by thorough and deliberate physical examination. With care, one can in these cases almost always arrive at a correct diagnosis.

13. To establish with certainty the diagnosis of appendicitis during pregnancy, it is necessary to exclude the presence of myalgia due to stretching of abdominal muscles, typhoid fever, ruptured or non-ruptured tubal pregnancy, cholecystitis, salpingitis, ovaritis, adnexitis, ovarian cyst with or without a twisted pedicle, rightsided pyelitis and ureteritis, fecal impaction, hepatic and nephritic colic. At times, any of the fore-mentioned conditions so closely resemble appendicitis as to cause diagnostic errors and operative mistakes.

14. The morbidity and mortality of appendicitis complicating pregnancy and the puerperium are the morbidity and mortality of delay in applying efficient surgical treatment. The initial symptoms of the attack do not enable the clinician to foretell accurately how a given case will terminate. What is going to happen in ten, twenty or forty hours following the onset of appendicitis cannot be foreseen. When the condition is diagnosed and remedied early, the mortality is practically nil. Abscess formation may be forestalled by early diagnosis and late operation. The pregnant woman whose metabolism is good is a good subject for operative measures.

15. Prognosis is better for the mother if there be no interruption of pregnancy spontaneous and otherwise. The bad attacks cause abortions and abortion aggravates the illness. In the great majority of surgically treated there is no interruption of pregnancy and when it does occur it is not due directly to the operation. The interruption of pregnancy is not indicated. It aggravates the prognosis. The fetal prognosis is good in early operated cases.

16. The following prophylactic measures are sound and safe and are recommended for general adoption: (a) During the child-bearing age, recurrent attacks of pelvic pain, dysmenorrhea, menstrual and other pelvic disturbances unassociated with objective pelvic findings are not infrequently due to unrecognized appendicitis or sequelae thereof. In the presence of this etiological factor, the ablation of the appendix is indicated. (b) In laparotomies for conditions other than appendicitis, the appendix should be examined. Should it present any deviation from the normal, its removal is indicated. (c) During the child-bearing age, any woman who has had one or more attacks of appendicitis treated non-operatively should have her appendix removed so as to correct existing pathological conditions and prevent future attacks of appendicitis and complications incident thereto. True prophylaxis in a woman of child-bearing age who has had one or more well marked attacks of appendicitis is an interval operation. It goes without saying that constipation is to be avoided and that other hygienic precautions are to be observed.

17. A definite and accurate diagnosis of acute, chronic or recurrent appendicitis, irrespective of the stage of pregnancy, invariably calls for operation. The disease during pregnancy runs such a rapid destructive course that delay is hazardous. Operation should be early and immediate. A case may be rendered hopeless by hesitation and inaction. Temporizing methods are extremely dangerous.

18. Treat appendicitis in the pregnant female as you treat it in the non-pregnant. Every pregnant woman who is a subject of appendicitis should be operated on just as soon as the diagnosis is made, whether the attack is the first, second or third.

The unusual risks of leaving a diseased appendix in the abdominal cavity are much increased by the pregnant state and the evil consequences of another attack, i.e., gangrene or perforation will be correspondingly greater. The danger of recurrence in the later months of pregnancy and in the child-bed period calls for operation preferably during the attack. If the patient is not seen in time, one will do the next best thing, an interval operation during the pregnancy. Pregnancy is an additional indication for operation in cases of appendicitis.

19. In inflammatory disease of the appendix, the ideal operation is an appendectomy. In some cases, however, one has to be content with incision, evacuation and drainage of an appendiceal abscess. Exceptionally drainage of abscesses in Douglas' pouch may be effected through the vagina or rectum. Pus should be evacuated irrespective of uterine contents, and irrespective of its location.

20. It is well to keep in mind that for an appendectomy the median incision is contradicted in the later months of pregnancy, that it is best to avoid or to reduce to a minimum the manipulations of the uterus; opiates are indicated in the after treatment. Labor when it occurs shortly after a laparotomy is not to be unduly prolonged; it may have to be assisted.

---

**Human Rabies.** Grenier de Cardenal, Legrand and Benoit refer to 6 convulsive cases with death in 48 hours reported by Jourdan and Marchand and one of paralytic type with death in 5 days. The first three authors report three other cases, one of slow development, without history of dog bite but probably due to horse bite in military service, death occurring in syncope in three days; a case in woman, dying in 48 hours and a fulminant case in a girl of 12 with death in three hours. In the first of these three cases, the primary diagnosis was angina, diagnosis being made from the typic, literal hydrophobia usually denied by modern writers.

